# A protocol for a scoping review of variations among psychedelic interventions for psychological suffering associated with the end-of-life

**DOI:** 10.1371/journal.pone.0318343

**Published:** 2025-05-06

**Authors:** Sarah Kratina, Carol Strike, Robert Schwartz, Ayah Nayfeh, Sydney Jopling, Chris Lo, Brian Rush

**Affiliations:** 1 Institute of Health Policy, Management and Evaluation, Dalla Lana School of Public Health, University of Toronto, Toronto, Ontario, Canada; 2 Department of Psychiatry, Cumming School of Medicine, Mathison Centre for Mental Health Research and Education, Hotchkiss Brain Institute, University of Calgary, Calgary, Alberta, Canada; 3 Public Health Sciences, Dalla Lana School of Public Health, University of Toronto, Toronto, Ontario, Canada; 4 Institute for Mental Health Policy Research, Centre for Addiction and Mental Health, Toronto, Ontario, Canada; 5 Independent Researcher, Toronto, Ontario, Canada; 6 Department of Psychiatry, Temerty Faculty of Medicine, University of Toronto, Toronto, Ontario, Canada; 7 School of Social and Health Sciences, Tropical Futures Institute, James Cook University, Singapore, Singapore; Kazimierz Wielki University in Bydgoszcz: Uniwersytet Kazimierza Wielkiego w Bydgoszczy, POLAND

## Abstract

Psychedelic substances are increasingly recognized for their therapeutic potential to ease psychological suffering linked to end-of-life issues. However, amid renewed scientific and public interest, policy remains restrictive. Existing reviews have made progress in synthesizing the results of studies of psychedelic interventions, especially psilocybin, and particularly with regard to their outcomes related to anxiety and depression, long-term effects and safety. Despite this progress, a wide range of both substances (such as ayahuasca, psilocybin, ketamine) and therapeutic approaches (such as psychedelics alone, or psychotherapy assisted with a psychedelic) in the use of psychedelic interventions specifically for end-of-life populations, has not been adequately covered by reviews to date. The aim of this scoping review is to identify and learn from the variety of psychedelic substances and therapeutic approaches that exists within the research on therapeutic psychedelic interventions reported in populations coping with psychological suffering associated with life-threatening illness and the end of life itself. We will follow Arksey and O’Malley’s (2005) framework for scoping reviews while incorporating updated methodological guidance. The Preferred Reporting Items for Systematic Review and Meta-Analyses extension for scoping reviews (PRISMA-ScR) guideline will be used to organize the search and identification of research focusing on psychedelic interventions, psychological suffering, and end-of-life issues. Health science databases such as Medline, Embase, APA PsychINFO, and CINAHL will be searched. The search will be limited to empirical published data on ‘end-of-life’, ‘psychedelics’, and ‘psychological suffering’. Data extracted from selected studies will cover intervention details, participant characteristics, measured outcomes, and theorised mechanisms. The insights gained from this review will be used to inform future research and discussions on how psychedelics can be integrated into care strategies for populations coping with end-of-life concerns. This scoping review does not require ethics approval.

## Introduction

Psychedelic substances are increasingly being recognized for their potential to alleviate psychological suffering in populations coping with life-threatening illness and the end-of-life [1]. After a decades-long gap between the 1980s and the early 2000s, scientific and clinical interest has been renewed in the healing potential of therapeutic psychedelic interventions (TPIs). Psychedelics, namely lysergic acid diethylamide (LSD) and mescaline, were initially studied to treat alcoholism, neuroses and as tools to better understand the causes and treatment of schizophrenia [2–6].

The term ‘psychedelic’ encompasses a diverse range of psychoactive drugs. These substances include ‘classic psychedelics,’ compounds that act on serotonin receptors, especially 5-HT2A, and include LSD, N,N-Dipropyltryptamine (DPT), psilocybin, mescaline, dimethyltryptamine (DMT) and also ayahuasca. Other substances considered ‘quasi-psychedelics’ have come to belong within the psychedelic term because they produce profound changes in perception, mood and cognition, and may be accompanied by visual hallucinations, but differ in the brain receptors they affect, include ibogaine, ketamine and the amphetamine 3,4-Methylenedioxymethamphetamine (MDMA).

Initial research on LSD in the context of terminal illnesses began in the early 1960s, paving the way for continued research of TPIs in populations more broadly coping with end-of-life concerns [7]. This includes participants diagnosed with life-threatening conditions who may expect prolonged survival but remain at risk of early mortality (e.g., people coping with AIDS) [8]. More recently, the resurgence of interest in TPIs has been fueled by a series of pivotal open-label studies or randomized controlled trials on MDMA-assisted therapy, psilocybin-assisted therapy, and LSD-assisted therapy along with intranasal ketamine alone for the treatment of anxiety and/or depression [9–15]. This momentum has been further bolstered by policy shifts and drug approvals, notably approvals for intranasal esketamine (Spravato®) for the treatment of a depressive disorder [16,17]. Currently, a variety of TPIs exist to treat psychological suffering linked to end-of-life concerns. However, the landscape of these interventions and their individual components remains inadequately assessed.

Existing studies discuss the importance and significance of an individual’s mindset and environmental setting during a psychedelic experience in determining the intervention’s effectiveness [18]. Many researchers and clinicians hold the perspective that the psychedelic substance acts more as an adjunct to psychotherapy rather than a stand-alone drug intervention [19,20]. Understanding these factors is essential for the development of clinical protocols, to indicate priorities for future research, and inform policies that are conducive to the judicious implementation of psychedelic interventions within the context of life-threating illness [21].

### International policy context

In 2020, the state of Oregon in the US became the first to legalize psilocybin-assisted therapy for mental health concerns, including for the end-of-life, with Colorado being the second state to legalize treatment centers using psychedelics [22]. Many US states are revising their legal frameworks to de-prioritize the enforcement of laws prohibiting the possession and use of psychedelics by law enforcement [22]. However, despite these shifts, the medical use of psychedelics is progressing more slowly. Most recently, on August 9^th^, 2024 the US Food and Drug Administration (FDA) declined to approve Lykos Therapeutics’ therapy combining MDMA with psychotherapy to treat post-traumatic stress disorder (PTSD).

In Canada, those facing a life-threatening condition have primarily two avenues to access psychedelic therapies. The first is through clinical trials and the second is via Health Canada’s Special Access Program (SAP) [23]. The discretion offered by the SAP has led to the therapeutic use of psilocybin for end-of-life distress.

On July 1, 2023, the Therapeutic Goods Administration authorized Australian psychiatrists to prescribe MDMA and psilocybin for treatment-resistant depression and post-traumatic stress disorder within controlled clinical care [24], although at the time of this writing, few have been treated. In Europe, psychedelic therapies are not approved, but the European Medicines Agency is supporting clinical trials with MDMA, LSD and psilocybin while creating a multidisciplinary advisory body to guide regulators on best practices with psychedelics [25]. Notably, in 2023, the Dutch government took a significant step by establishing an independent state commission to evaluate the safety and effectiveness of the medical use of MDMA [26]. The policy changes observed across Canada, the U.S., Australia, and Europe reflect a broader global trend towards a more nuanced, evidence-based understanding of psychedelics. The shift is moving away from strict criminalization and there is growing interest in their therapeutic value.

### Reviews of therapeutic psychedelic interventions for those diagnosed with a life-threatening illness

Prior reviews of TPIs to address end-of-life concerns have tended to focus on the effectiveness of treatment using meta-analysis, traditional systematic review, and narrative approaches [27–32]. In general, meta-analyses across clinical populations have found that medium to large effects (e.g., effect sizes from 0.6–1.2, Hedges g) on primary outcomes such as anxiety, depression and post-traumatic stress disorder psychological outcomes are possible [33,34]. The meta-analyses synthesize findings from studies across diverse populations. This includes not only participants with a life-threatening illness, as was solely examined in one meta-analysis [35], but also those with various conditions such as autism, chronic post-traumatic stress disorder, and treatment-resistant major unipolar depressive disorder.

The systematic reviews examined the long-term effects (such as lasting changes in anxiety, depression and spirituality) [36], safety [37,38] and selected mechanisms that may fall along disciplinary lines [39–43]. These reviews suggest the variability of psychedelic interventions within the clinical trials conducted, e.g., [44–46]. This variability includes the inclusion of TPIs with or without music, such as single-dose psilocybin in conjunction with psychotherapy of an unspecified type, psilocybin and adjunctive psychotherapy with an emphasis on the process of meaning making and two LSD-assisted psychotherapy sessions of an unspecified type. Conclusions from these reviews are based on combining the effects across different psychedelics (e.g., ayahuasca, psilocybin, LSD), interventional models and participant groups. Outcomes relating to depression and anxiety may show wide variation in operationalization of the construct (e.g., ranging from social anxiety in autistic adults, treatment-resistant major depressive disorder in adults, to anxiety and depressive symptoms associated with end-of-life issues) [34,47,48].

Recent research [49] has indicated that existing reviews often do not adequately characterize the psychosocial components integral to psychedelic interventions, beyond the specific substance administered. They overlook key factors such as the type and extent of accompanying psychotherapy; the qualifications of the therapist or guide; and the setting in which the therapy occurs. These elements are critical to identify and describe because they can influence treatment outcomes by shaping therapeutic alliance, patient comfort, expectations and engagement. Additional aesthetic elements like music, eyeshades, or art are important [31,32] and may not be adequately described. Aesthetic elements contribute to creating a supportive and immersive environment, potentially enhancing emotional processing, and the overall therapeutic experience. Across the existing reviews, there is agreement that the progress of research has been restricted by stringent governmental regulations on controlled substances, e.g., [37,43]. Researchers advocate for more rigorous studies backed by government funding [29], and more extensive study of non-cancer populations coping with life-threatening illness, e.g., [8].

### Extending the scope: Addressing gaps and clarifying contexts

Existing reviews have made progress in synthesizing the results of studies of TPIs for participants with a life-threatening illness, particularly psilocybin, and with regard to outcomes related to anxiety and depression, long-term effects and safety. Despite this progress, reviews do not adequately characterize the range of therapeutic approaches and psychedelic substances.

The purpose of this review is to scope the variation in TPIs addressing psychological suffering associated with end-of-life concerns. This kind of distress arises from coping with a foreshortened future in response to a life-threatening illness. Typically, the literature considers a life-threatening illness to be a terminal disease, such as advanced cancer. However, a life-threatening illness need not be in the terminal stages to activate concerns about mortality and the end of life. For example, patients with long-term HIV/AIDS can experience distress from a psychologically foreshortened future [8]. Further, this distress need not only be in response to physical disease but can include mental illness in which survival is poor, such as with patients with life-threatening suicidality [50–52].

## Methods

This scoping review method was selected because it is well-suited to map the breadth of existing literature, particularly in this field where studies are diverse and methods heterogenous. The review will be based on the framework proposed by Arksey and O’Malley in their seminal paper “Scoping studies: Towards a methodological framework” [53] while incorporating updated methodological guidance [54]. This approach to scoping reviews is comprised of five key stages: (1) identifying the research question(s); (2) identifying relevant studies; (3) study selection; (4) charting the data; and (5) collating, summarizing, and reporting the results. The Preferred Reporting Items for Systematic Review and Meta-Analyses extension for scoping reviews (PRISMA-ScR) guideline will be used to organize the search and identify the research for the scoping review [55].

### Stage 1: Identifying the research questions

The primary research question is: What is known about how empirical studies vary in the TPI literature in populations coping with life-threatening illness? This question was examined by focusing on four aspects of variation: (1) study characteristics (e.g., time and place of study, inclusion/exclusion criteria); (2) interventional characteristics (e.g., type of substance, clinical environment); (3) outcomes measures; and (4) postulated mechanisms by which TPIs were thought to achieve their effects.

### Stage 2: Identifying relevant studies

The term ‘end of life’ will be selected for use in this research effort to capture the experience of confronting one’s mortality, among population groups coping with either an acute or chronic illness that may be considered a life-threatening illness, life-limiting condition, or terminal illness. The overarching problem or indication of interest will be ‘psychological suffering’. This term will be selected to encompass the multifaceted negative experiences that can occur when diagnosed with a life-threatening illness, beyond the indications of anxiety and depression, and includes existential distress, hopelessness, demoralization, meaninglessness, and loss of dignity. TPIs will be explored, specifically, psychedelics that were investigated within a research context for those diagnosed with an end-of-life issue. The term ‘therapeutic psychedelic interventions’ can include psychedelics as a stand-alone intervention without psychotherapeutic support or integrative follow-up, and psychedelics administered as an adjunct to a psychotherapeutic modality.

A search strategy will be developed by S.K. and conducted in four electronic databases from inception to October 27^th^, 2023: Medline, Embase, APA PsychINFO, and CINAHL. The following concepts will be searched in combination: ‘end-of-life’, ‘psychedelics’, and ‘psychological suffering’. The search strategy will not be piloted. After the search strategy is run in each of the databases, S.K. will review the retrieved articles to ensure that seminal research studies were included. A brief outline of the search strategy for a database is provided in Table 1. Psychedelics and end-of-life issues are the two essential search concepts that increase sensitivity to retrieve all relevant research. The concept of psychological suffering adds specificity. Concepts will be searched with the appropriate inclusion of synonyms. The search will be limited to journal articles. The reference lists from included papers will be searched along with forward citation searching using Google Scholar and backward citation of the references of included articles.

**Table 1:** Example search terms used in the search strategy for Embase.

	Example Search Terms
1	exp ketamine/
2	exp psilocybin/
3	exp midomafetamine/
4	exp esketamine/
5	exp ayahuasca/
6	exp psilocybine/
7	exp lysergide/
8	exp psychedelic agent/
9	exp psychedelic therapy/
10	exp ibogaine/
11	exp mescaline/
12	exp n,n dimethyltryptamine/
13	1 or 2 or 3 or 4 or 5 or 6 or 7 or 8 or 9 or 10 or 11 or 12
14	(ketamine or esketamine or ayahuasca or psilocyb* or peyote or MDMA or 3,4-Methylenedioxymethamphetamine or DMT or DPT or N,N,dipropyltryptamine or ‘lysergic acid diethyl amide’ or LSD or psychedel* or hallucinogen* or ‘psychedelic assisted psychotherapy’ or ‘psychedelic assisted therapy’ or ‘psychedelic therapy’ or entheogen or mescaline or ibogaine).ti,ab,kw.
15	13 or 14
16	exp psychedelic therapy/
17	exp terminal care/
18	exp terminal disease/
19	(‘end of life’ or cancer or ‘palliative care’ or palliative or ‘life threatning illness’ or ‘life threatening disease’ or ‘terminal disease’ or ‘terminal care’ or ‘palliative therapy’).ti,ab,kw.
20	16 or 17 or 18 or 19
21	exp depression/
22	exp anxiety/
23	exp distress syndrome/
24	exp demoralization/
25	(‘existential distress’ or ‘existential suffering’ or ‘death distress’ or hopelessness or anxiety or depression or demoralization or ‘psychological suffering’ or ‘psychological distress’).ti,ab,kw.
26	21 or 22 or 23 or 24 or 25
27	15 or 20 or 26
28	limit 27 to “review”
29	27 not 28

### Stage 3: Study selection: Inclusion and exclusion criteria

#### Types of participants.

This review will include articles in which adult participants (age ≥ 18 years) provided informed consent to participate in research examining the effects of psychedelic interventions for the purpose of alleviating psychological suffering and its affiliated constructs (e.g., depression, anxiety, hopelessness) associated with end-of-life issues.

#### Types of interventions.

This review will include articles describing the effects of psychedelics, i.e., ketamine, psilocybin, ayahuasca, 3,4-Methylenedioxymethamphetamine (MDMA), N,N-Dipropyltryptamine (DPT), lysergic acid diethylamide (LSD), ibogaine, peyote, and mescaline, used to manage concerns related to a life-threatening illness, using any dosage, or any type of adjunct therapy (e.g., psychotherapy, music therapy).

#### Types of data sources.

This review will include publications of primary empirical studies of any data type (quantitative, qualitative, mixed methods) and design (e.g., clinical trials, observational studies, case studies), published in peer-reviewed research journals. The language will be restricted to English and French because they are Canada’s official languages, as well as Spanish and Portuguese due to the historical and current Latin and Central American contributions to the study of psychedelics within Indigenous Peoples. This review imposed no restrictions on publication date, or type of methodology. The articles must be full-text empirical publications; abstracts, poster presentations and dissertations will be excluded.

The selection procedure will consist of two related steps: (1) identification of articles for inclusion by title and abstract screening and (2) full-text screening of those identified as potentially eligible in step (1). The screening for eligibility of retrieved articles will be done using Covidence [56]. Table 2 describes the concepts that will be used as part of the identification and screening process. The article identification and screening process will be based on the Preferred Reporting Items for Systematic Review and Meta-Analyses (PRISMA) extension for scoping review flow diagrams [55]. Duplicates will be removed using Covidence. Two reviewers will independently screen and assess the eligibility of retrieved articles. Both reviewers will screen the titles and abstracts of all results retrieved from the four databases. After screening all titles and abstracts, the full text of eligible articles will be independently assessed to ensure that inclusion criteria are met. Discrepancies will be resolved through discussion between the reviewers.

**Table 2:** Population, intervention, and outcome framework.

Concept 1: Population	Concept 2: Intervention	Concept 3: Outcome
Adult participants (age ≥ 18 years) coping with life-threatening illness and the end-of-life. Specifically, those who are diagnosed with either an acute or chronic illness that may be considered life-threatening, life-limiting, or terminal.	Therapeutic psychedelic interventions encompass psychedelics that are an adjunct to psychotherapy. It also includes psychedelics as a stand-alone intervention without psychotherapeutic support or integrative follow-up. Psychedelics is a term applied to various psychoactive drugs that belong to different classes, which produce profound altered states of consciousness, affecting a person’s perception, mood and cognitive processes. These include lysergic acid diethylamide (LSD), ketamine, psilocybin, 3,4-Methylenedioxymethamphetamine (MDMA), and N,N-Dipropyltryptamine (DPT) and ayahuasca.	A multifaceted experience of psychological suffering associated with end-of-life issues that includes but is not limited to anxiety and depression, largely characterized by existential distress, demoralization, meaninglessness and loss of dignity.

### Stage 4: Charting the data

The same research team members will extract data from the included articles using a structured form. The data extraction form was structured by the guidelines set out in the Joanna Briggs Institute (JBI) Manual for Evidence Synthesis, ensuring a structured and systematic approach to capturing relevant data [53]. This form has been designed to extract detailed descriptive data that will aid in providing a comprehensive understanding of topics and quantifiable data. The form also allows for the extraction of descriptive data to encompass more contextual and qualitative information to be included.

Three team members will independently chart data from the first five articles to pilot-test the data extraction form and ensure consistent and comprehensive data collection. The team members will compare and discuss the extracted data and consider whether modification of the extraction form will be necessary to ensure that the data collected meets the review’s aim. Disagreements among the three team members charting the data will initially be resolved through collective discussion, if consensus cannot be reached, a senior research team member will provide the final decision. The data will be recorded and saved in a Google Drive Excel sheet. As is standard practice for scoping reviews, the quality of evidence will not be appraised.

### Stage 5: Collating, summarizing and reporting the results

This scoping review will summarize the evidence regarding the variation of TPIs that have been used to alleviate psychological suffering associated with end-of-life issues. All the data will be organized in a data extraction form. The relevant study and intervention characteristics in each included article will be descriptively synthesized. No datasets were generated or analyzed in this scoping review protocol. Instructions concerning data availability will be provided in the scoping review study that publishes the empirical results.

## Limitations

This scoping review used a systematic approach designed to broadly map the research landscape of TPIs in populations with end-of-life issues. The studies that will be included in this scoping review will likely be heterogeneous in terms of study designs, methodologies, and outcome measures, which may impact the ability to draw generalized conclusions. A detailed quality assessment of the included studies will not be included, potentially affecting the reliability of the findings.

## Conclusion

This scoping review will comprehensively explore the research field of TPIs for individuals coping with life-threatening illness, providing insights that will contribute to the discussion and understanding of the variation among TPIs for psychological suffering linked to end-of-life concerns. By identifying and describing the implication of the heterogeneity of study designs, and outcome measures, this review will inform the development or incorporation of relevant measurement tools. Furthermore, the description of psychedelic interventional approaches studied in the empirical literature will serve as a foundation for identifying strengths and gaps in knowledge. The review’s insights will contribute to the development of future research priorities and inform the shaping of clinical protocols and policies for psychedelic applications.
